# Impact of Polymerization Technique and ZrO_2_ Nanoparticle Addition on the Fracture Load of Interim Implant-Supported Fixed Cantilevered Prostheses in Comparison to CAD/CAM Material

**DOI:** 10.3390/dj10060102

**Published:** 2022-06-08

**Authors:** Faris A. Alshahrani, Shorouq Khalid Hamid, Lujain Ali Alghamdi, Firas K. Alqarawi, Yousif A. Al-Dulaijan, Hamad S. AlRumaih, Haidar Alalawi, Maram A. Al Ghamdi, Fawaz Alzoubi, Mohammed M. Gad

**Affiliations:** 1 Department of Substitutive Dental Sciences, College of Dentistry, Imam Abdulrahman Bin Faisal University, P.O. Box 1982, Dammam 31441, Saudi Arabia; fkalqarawi@iau.edu.sa (F.K.A.); yaaldulaijan@iau.edu.sa (Y.A.A.-D.); hsalrumaih@iau.edu.sa (H.S.A.); haalalawi@iau.edu.sa (H.A.); maalghamdi@iau.edu.sa (M.A.A.G.); mmjad@iau.edu.sa (M.M.G.); 2 Ministry of Health, Dammam 31441, Saudi Arabia; shorouqh@moh.gov.sa (S.K.H.); lualghamdi@moh.gov.sa (L.A.A.); 3 Department of General Dental Practice, Faculty of Dentistry, Kuwait University, Kuwait City P.O. Box 24923, Kuwait; fawaz.alzoubi@ku.edu.kw

**Keywords:** ZrO_2_ nanoparticles, fracture load, cantilever, PMMA, polymerization technique

## Abstract

ZrO_2_ nanoparticles (ZNPs) have excellent physical properties. This study investigated the fracture load of implant-supported, fixed cantilevered prosthesis materials, reinforced with ZNPs and various polymerization techniques, compared with conventional and CAD/CAM materials. Sixty specimens were made from two CAD/CAM; milled (MIL) (Ceramill TEMP); and 3D-printed (NextDent Denture 3D+). Conventional heat-polymerized acrylic resin was used to fabricate the other specimens, which were grouped according to their polymerization technique: conventionally (HP) and autoclave-polymerized (AP); conventionally cured and reinforced with 5 wt% ZNPs (HPZNP); and autoclave reinforced with 5 wt% ZNPs (APZNP). The specimens were thermocycled (5000 cycles/30 s dwell time). Each specimen was subjected to static vertical loading (1 mm/min) using a universal Instron testing machine until fracture. Scanning electron microscopy was used for fracture surface analyses. The ANOVA showed significant fracture load differences between all the tested groups (*p* = 0.001). The Tukey post hoc tests indicated a significant difference in fracture load between all tested groups (*p* ˂ 0.001) except HP vs. HPZNP and AP vs. MIL. APZNP had the lowest mean fracture load value (380.7 ± 52.8 N), while MIL had the highest (926.6 ± 82.8 N). The CAD/CAM materials exhibited the highest fracture load values, indicating that they could be used in long-term interim prostheses. Autoclave polymerization improved fracture load performance, whereas ZrO_2_ nanoparticles decreased the fracture load performance of cantilevered prostheses.

## 1. Introduction

The treatment of completely edentulous patients in association with severe arch resorption by using conventional complete dentures poses extreme challenges in terms of retention, stability, and support [1]. The use of dental implants for treating complete edentulism is well documented in the literature, having a successful long-term outcome, enhancing oral function, and improving psychosocial effects [2]. In cases where local residual edentulous ridge conditions hinder the probability of implant placement, adding a cantilever extension to the implant-supported prosthesis may be considered a viable option [3]. However, the cantilever extension needs to be able to withstand the masticatory load and bending forces of the oral cavity [4]. The fracture resistance of interim materials is particularly significant in the treatment of cantilevered segments, immediate loading, and long spans when the prosthesis is used over a long period or if the patient has parafunctional habits [5].

Although computer aided design/computer aided manufacturer (CAD/CAM) has significantly superior mechanical characteristics, ease of use, and quality, its initial high cost was deemed to be a major drawback [6]. In 2017, Al-Dharrab compared the surface hardness, flexural strength, and modulus between heat-polymerized PMMA and CAD/CAM-prepolymerized resin blocks. Heat-polymerized PMMA showed significantly higher flexural strength, while CAD/CAM resin material had a significantly higher surface hardness and flexural modulus [7].

Polymerization techniques have been modified over the years to advance the mechanical and physical characteristics of resin materials. PMMA undergoes a variety of polymerization techniques, including heat, light, autoclave, and microwave energy [8]. The heat polymerization technique, also known as a hot water bath, is the conventional method for PMMA polymerization [9]. The heat polymerization technique can be altered using a range of time and temperature parameters, which can lead to incomplete conversions of monomers, resulting in an increased amount of residual monomer that behaves as a plasticizer and disturbs the mechanical and physical characteristics of acrylic resins [8]. An autoclave polymerization technique has since been suggested as an alternative to conventional water bathing and has been shown to enhance the mechanical properties of PMMA [8,10]. It significantly decreases the amount of residual monomer formation and increases the mechanical properties of the material [8,10].

Nanomaterials have seen tremendous advances, being the subject of a variety of research areas and having many favorable applications in biomedical science and nanomedicine due to their superior chemical, physical, and antimicrobial properties [11]. ZrO_2_ nanoparticles (ZNPs) have garnered much attention for their technological and scientific potential [12]. This nanobiotechnology has potential for wide-ranging biological application because of its high strength and low thermal conductivity. Heat-insulating properties and pliability are characteristics which have made ZNPs extremely useful in the medical field, especially within dentistry, for use in orthopedic implant materials and dental filling composites [13].

Gowri et al. evaluated the hybrid effect of zirconia nanocomposites with aluminum borate whiskers on the mechanical characteristics of denture base resins. Their results showed a significant improvement in the mechanical characteristics of these resins [12]. In 2018, Gad et al. assessed the impact of 2.5, 5, and 7.5% ZNP concentration additions on the optical and tensile properties of PMMA. They concluded that the addition of ZNPs to heat-polymerized PMMA increases its tensile strength in a direct relationship when compared to the control group [14]. In the tested PMMA/nano-ZrO_2_ materials, flexural strength increased when the ZNPs ranged from 0.5 wt% to 5 wt%; above these concentrations, there was no observed effect on flexural strength [15,16]. Therefore, we chose low ZNP concentrations due to the positive effect this has on flexural strength. Additionally, these lower concentrations allow for the even distribution of added nanoparticles within a polymer matrix [16].

The mechanical strength of implant-supported, fixed cantilevered prostheses (ISF-CP) is considered an important feature that impacts the long-term success of interim dental prostheses. To our knowledge, there have been no previous studies on the effect of ZNP reinforcement on the fracture load of ISF-CP. The aim of this in vitro study was to assess the fracture load of ISF-CP materials reinforced with ZNPs via different polymerization techniques and compare them to CAD/CAM materials. The null hypothesis stated that the addition of ZNPs and autoclave polymerization would have no significance on the fracture load of ISF-CP materials.

## 2. Materials and Methods

**Figure 1:**
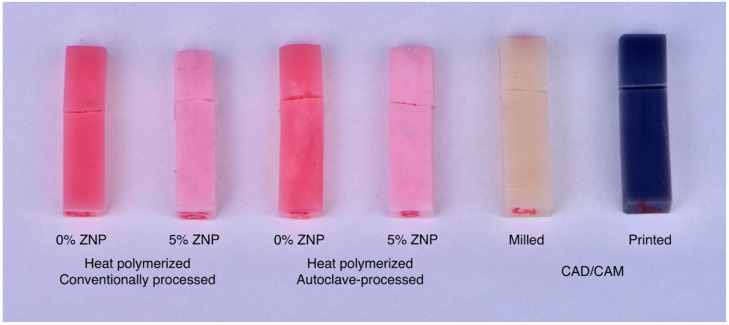
Specimens were fabricated and distributed into six groups according to their material, polymerization technique, and ZNP concentration.

A total of 60 specimens were fabricated and distributed into six groups according to their material, polymerization technique, and ZNP concentration: unmodified heat-polymerized as control (HP), a ZNP-modified heat-polymerized group (HPZNP), an autoclave-polymerized group (AP), a ZNP-modified autoclave-polymerized group (APZNP), a CAD/CAM milled group (MIL), and a three-dimensional (3D) printed group (Figure 1).

### 2.1. PMMA/ZrO_2_NP Mixture Preparation

ZNPs (99.9% < 100 nm, 1314-23-4; Shanghai Richem International Co., Ltd., Shanghai, China) were treated with a silane coupling agent [3-(trimethoxysilyl) propyl methacrylate] (Shanghai Richem International Co., Ltd., Shanghai, China) in order to enhance ZNP adhesion to the acrylic resin matrix, as explained in previous research [10,15]. The treated ZNPs were weighed using an electronic balance and added to heat-polymerized PMMA powder (BMS 014 powder; BMS Dental, Capannoli, Italy) with a concentration of 5%. Then, it was mixed by an electric mixer for 30 min to reach a uniform particle distribution and homogenous color.

### 2.2. Heat-Polymerized Acrylic Resin Specimens

Rectangular-shaped metal negative molds were fabricated with internal dimensions of 7 mm in buccolingual width, 8 mm in occlusocervical thickness, and 30 mm in length (8 × 7 × 30 mm^3^) [5]. Wax specimens were obtained through metal molds and then invested in a metal flask and type III dental stone. A wax elimination machine was used to remove the wax. The separating medium was spread throughout the stone’s surfaces while it was still warm. The acrylic resin (with and without ZNP reinforcement) monomer/powder ratio was measured and then mixed as instructed by the manufacturer. At the dough stage, the mixture was packed under pressure into previously prepared molds. The flasks were then stored in flask clamps for 1 h prior to processing.

For the conventional polymerization technique, flasks were processed by a thermal curing unit (KaVo Elektrotechnisches Werk GmbH, Biberach, Germany) at 74 °C for 8 h, then at 100 °C for 1 h. For the autoclave polymerization technique, flasks were processed using an autoclave under 3 atm pressure for 60 °C for 30 min and then 130 °C for 20 min [10]. Following complete polymerization, flasks were cooled to room temperature and deflasked afterward. Specimen finishing was performed using a tungsten carbide bur, and then polished by a coarse grain followed by a fine grain cylindrical rubber top bur (Super Acrylic Polisher; Long Dental, Wilmington, NC, USA). A digital caliper with 0.01 mm accuracy was utilized to assess the specimens’ dimensions. Afterward, specimens were stored in distilled water at 37 °C for 7 days [17].

### 2.3. CAD/CAM Specimen Preparation

Specimens were designed by employing open-source CAD software standard tessellation language (STL) files (123D design, Autodesk, version 2.2.14, San Rafael, CA, USA) prior to manufacturing. For milled specimens, Ceramill TEMP CAD/CAM high-density polymers (HDPs) (Ceramill TEMP light 71 L20nm, Amann Girrbach AG, Koblach, Austria) were used to fabricate rectangular shapes with the dimensions of 8 × 7 × 30 mm^3^. HDPs were CAD (Ceramill mind, Amann Girrbach AG, Koblach, Austria) by STL files and milled using a CAM milling machine (Ceramill matik, Amann Girrbach AG, Koblach, Austria). According to the manufacturer’s instructions, a 5-axis milling process with 2.5 mm maximum and 1 mm minimum diameter burs was used to fabricate more precise details, under wet conditions to avoid overheating [5,18].

3D-printed specimens (8 × 7 × 30 mm^3^) were fabricated utilizing an acrylate ester-based resin (NextDent Denture 3D+, 3D systems, Vertex Dental B.V., Soesterberg, The Netherlands) and a 3D printer (NextDent 5100, 3D systems, Vertex Dental B.V., Soesterberg, The Netherlands) at a 90° orientation, layer-by-layer (50 μm/layer), in a straight-lined pattern [17]. Following initial printing, the specimens were cleaned in an isopropyl alcohol solution as per the manufacturer’s recommendations. For postpolymerization, a UV light postcuring machine was used (LC-3DPrint Box, NextDent, Vertex Dental B.V., Soesterberg, The Netherlands) for 10 min. Afterwards, a low-speed rotary device (5000 rpm) was used to remove the specimens’ support structures, with the specimens then polished before testing [19]. Specimen thermal cycling was performed 5000 times (5 °C to 55 °C) in a water bath with a 30 s dwell time using a thermal cycling machine (Thermocycling TC-4, SD Mechatronik GmbH, Feldkirchen-Westerham, Germany) [20], simulating 6 months of clinical use.

### 2.4. Specimen Testing

**Figure 2:**
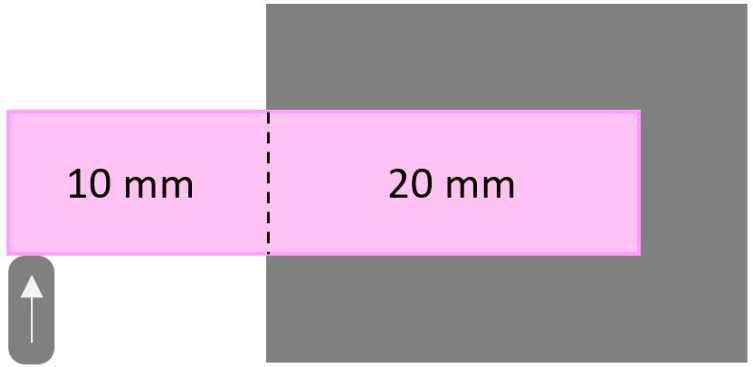
Illustrated diagram for loaded specimen with indenter location.

The specimens were tested immediately after their removal from the storage container to be tested under wet conditions. To acquire a 10 mm cantilever, the specimens were fixed using a clamp at the initial 20 mm measurement. Prior to load application, the load frame was orientated to contact the specimen 2 mm from its end (Figure 2). A universal testing machine (ElectroPuls®; Instron, Norwood, MA, USA) and a biaxial servohydraulic load frame (5 kN) were utilized to apply static loading at a 1 mm/min crosshead speed [14]. The load was applied vertically to the specimen until a fracture occurred, at which point the maximum fracture load values were registered [5]. Scanning electron microscopy (SEM) (JSM-IT200 InTouchScope™ Scanning Electron Microscope, JEOL Ltd. Akishima, Tokyo, JAPAN ) was utilized to evaluate each specimen’s fractured surface features (topography). To observe the essential features, specimens were sputter coated with gold (Quorum, Q150R ES, Laughton, UK), and images were acquired at several magnifications (500×, 1000×, and 2000×) [14].

### 2.5. Statistical Analysis

For data entry and analysis, IBM’s Statistical Package for Social Sciences (SPSS v. 23, IBM, Armonk, USA) was used. Normality of data was tested by the Shapiro–Wilk test, and nonsignificant *p* values (*p* > 0.05) proved that the data were normally distributed. Hence, parametric tests were used for the analysis. To investigate the variance in averages between groups, a one-way ANOVA was employed, and then a Tukey’s post hoc test was utilized for pair-wise comparisons of the means. *p* values ≤ 0.05 were considered statistically significant.

## 3. Results

According to the one-way ANOVA tests, there were statistically significant differences in fracture load values between all tested and control groups (F = 75.351, *p* ˂ 0.001). The highest fracture load values were recorded in the MIL group (926.6 ± 82.8 N), followed by the 3D (739.4 ± 58.8 N), AP (744.0 ± 76.1 N), and control (635.5 ± 43.4 N) groups. The HPZNP and APZNP groups showed the lowest fracture load values of 583.4 ± 58.1 N and 380.7 ± 52.8 N, respectively. As shown in Table 1, according to Tukey’s post hoc tests, the pairwise comparison indicated a significant difference in the fracture load values between all tested groups (*p* ˂ 0.001) except HP vs. HPZNP (*p* = 0.512) and AP vs. MIL (*p* = 1.000).

### SEM Analysis

**Figure 3:**
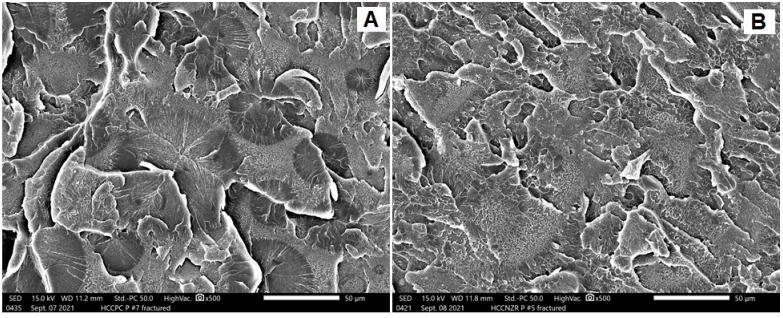
Representative SEM images for the fractured surfaces of (**A**) HP and (**B**) HPZNP.

**Figure 4:**
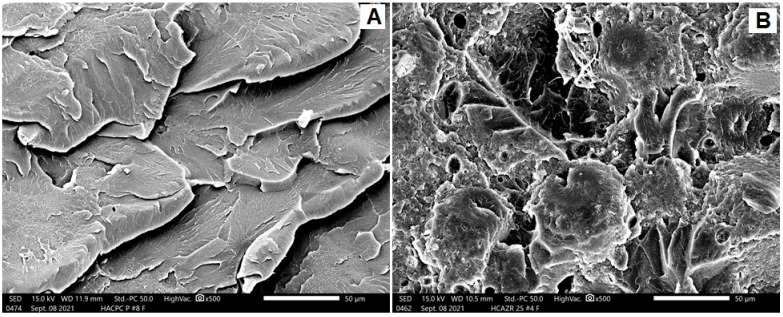
Representative SEM images for the fractured surfaces of (**A**) AP and (**B**) APZNP.

**Figure 5:**
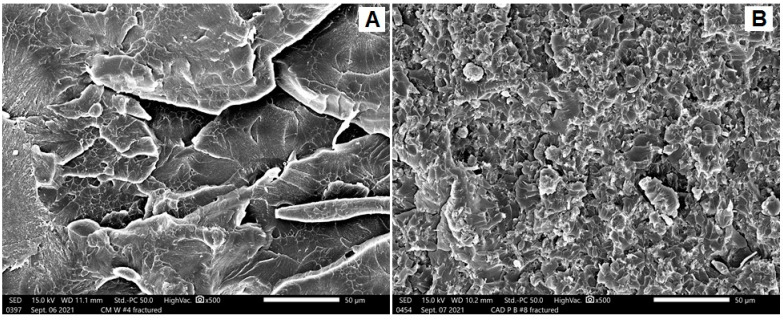
Representative SEM images for the fractured surfaces of (**A**) MIL and (**B**) 3D-printed.

Three modes of fractures were noted: ductile fracture (more irregularities and lamellae), brittle fracture (smooth surfaces with an absence of lamellae), and intermediate fracture (in-between features). Figure 3, Figure 4 and Figure 5 represent SEM images with a ×500 magnification, showing the topography of the fractured surfaces. For conventionally heat-polymerized PMMA, irregular surfaces with multiple steps and lamellae were observed (Figure 3A). With the addition of ZNPs, these features were less irregular, with very faint lamellae (Figure 3B). For pure autoclave specimens, uniform lamellae with sharp steps and deep grooves that accompanied each lamella were present (Figure 4A). These features dramatically changed into dominant, randomly oriented faint lamellae with a smooth surface when ZNPs were added (Figure 4B). The behavior of the CAD/CAM milled specimens (Figure 5A) resembled that of pure, conventionally heat-polymerized PMMA. The 3D-printed specimens showed unique characteristics, with irregular surfaces and small, curved, and faint lamellae absent of smooth background features (Figure 5B).

## 4. Discussion

A long-term interim prosthesis with a high fracture load value might be needed during the osseointegration period of an implanted material, especially in the presence of a cantilever. From this perspective, our study aimed to investigate the influence of ZNP addition and various polymerization techniques on the fracture load of ISF-CP in comparison to CAD/CAM materials. The results demonstrated a decreased fracture load value in the ZNP-reinforced groups. Furthermore, the MIL group had the highest mean fracture load value, whereas the autoclave polymerized with 5% ZNP group had the lowest fracture load value. Therefore, the null hypothesis (that ZNP reinforcement and autoclave polymerization have no significance on the fracture load of ISF-CP) was rejected.

Fracture is a common concern in ISF-CP, despite the advancements in restorative materials and procedures [21]. Therefore, it is essential to find a method to enhance the mechanical properties of a material. The present study results showed that the pure autoclave-polymerized specimens demonstrated a significant increase in fracture load value compared to the control group. As shown in the SEM analysis, the fractured surfaces of pure autoclave-polymerized specimens exhibited more ductile fractures compared to the control group. Increased crosslinking and decreased monomer formation could explain the notable enhancement in fracture load resistance due to the high heat treatment conditions [8,10]. Considering that monomer has a boiling point of 100.8 °C, during the conventional polymerization process, the monomer can evaporate and produce bubbles in the resin matrix, resulting in pore formation and deterioration in the PMMA mechanical characteristics. To avert this during autoclave polymerization, radical initiated polymerization is carried out, first at 60 °C to absorb all the free monomer, then at 100 °C or higher to crosslink the polymer [22]. Durkan et al. [10] investigated the impact of autoclave polymerization on the transverse strength of PMMA in comparison to the conventional water bath method; their results showed that the transverse strength of specimens significantly increased with autoclave polymerization. Moreover, Ayaz et al. [8] found that the hardness of PMMA statically increased with autoclave polymerization compared to the conventional technique.

In a systemic review performed by Leão et al. in 2020, 5% ZNP concentrations were recommended [23]. However, the results of the present study indicate that the addition of ZNPs to heat-polymerized conventionally-cured PMMA generated insignificant differences in fracture load resistance compared to the control group, which agrees with the SEM analysis findings. A possible explanation for this might be that the addition of ZNPs affects the degree of polymerization when added in large concentrations, which may weaken the interim prosthesis [23]. There are, however, other possible explanations, such as filler cluster formation, the degree of distribution of the additive inside the resin matrix, and the development of an interfacial layer between the resin matrix and the ZNPs [24]. In agreement with the current findings, a previous study performed by Zidan et al. [25], which investigated the mechanical characteristics of a ZrO_2_-impregnated PMMA nanocomposite, showed a significant decrease in fracture toughness and impact strength with increasing ZNP content. Another study, performed by Alhotan et al. [24], which tested fracture toughness when different ZNP concentrations were added to PMMA, found that the fracture toughness was insignificantly different from that of the control group, and dropped gradually when the ZNP concentrations were 5 and 7%. Despite the significant improvement in the fracture load of pure autoclave-polymerized specimens, reinforcement with ZNPs in the autoclave-polymerized PMMA was not found to enhance its fracture load. On the contrary, it showed a significant reduction in fracture load when compared to all groups. According to SEM analysis, its fractured surfaces showed a brittle fracture with smooth surface topography. A possible explanation for this might be the incomplete polymerization between particles, which can result in increased residual monomer formation that functions as a plasticizer, impacting the overall mechanical characteristics of the material [26].

According to the present results, the CAD/CAM groups had a significantly higher fracture load mean value, which is consistent with the results of a previous study [5]. A possible reason for this difference is the structure and manufacturing process of the material [27,28]. The CAD/CAM-milled group had the highest fracture load mean, which could have been caused by the elevated temperature and pressure polymerization, promoting the growth of longer polymer chains and decreasing the intermolecular distance with a lower amount of residual monomer [7,29].

3D-printed technology has been frequently used in the dental field to reduce cutting time and material waste. In the current study, the 3D-printed group had a lower fracture load mean than the milled group, which agrees with the findings from previous studies [30,31]. This could be explained by both the reactivity of the 3D-printed resin monomer and the curing conditions, which reduce the degree of double bond transformation. Another reason is the frail interlayer bonding between sequential printed layers [19,32]. Prpic et al. [31] stated that 3D-printed resin has mechanical properties inferior to those of milled and heat-polymerized resin. As found in SEM analysis, the specimen fracture surfaces in the milled group showed more irregularities and ductile fractures when compared to the 3D-printed group.

ISF-CP fabrication techniques affect fracture load resistance. Therefore, milled, 3D-printed, and autoclave-polymerized resins are more suitable for clinical use, having higher mechanical performance, especially CAD/CAM milled ISF-CP. An important strength of this study is its evaluation of different fabrication techniques for assessing the fracture load of ISF-CP, including heat-polymerized (conventional and autoclave) acrylic resin modified with ZNPs and CAD/CAM technology. Furthermore, artificial aging (thermocycling) was used (5000 cycles) to mimic 6 months of clinical use. During thermocycling, higher temperatures increase water entrance and elevate the plasticization effect, diminishing the mechanical properties of an acrylic resin [20]. ZNP-modified ISF-CP did not display fracture load improvement in the present study. However, investigating only one mechanical property (fracture load) against one concentration for each reinforcement material limited our findings. In addition, the mechanical tests were not carried out in a wet environment, meaning they are not representative of oral cavity conditions. Therefore, we recommend further investigation using different ZNP concentrations under simulated oral conditions, utilizing different brands of CAD/CAM ISF-CP. Moreover, additional analyses such as X-ray diffractometry, surface hardness, and Raman spectrometry are recommended for future studies.

## 5. Conclusions

The CAD/CAM material specimens had a significantly higher fracture load mean value, making the material suitable for long-term interim prosthesis. Autoclave polymerization improved fracture load resistance, whereas ZNPs decreased the fracture load resistance of cantilevered prostheses, prompting the need for further investigation using different ZNPs concentrations.

## Figures and Tables

**Table 1:** Mean, standard deviations, and significances of fracture load between groups.

	Heat Polymerized				CAD/CAM	
	Conventionally Processed		Autoclave-Processed			
	0% ZNP	5% ZNP	0% ZNP	5% ZNP	Milled	3D-Printed
**Mean (SD)**	635.5 (43.4) ^a^	583.4 (58.1) ^a^	744.0 (76.1) ^b^	380.7 (52.8)	926.6 (82.8)	739.4 (58.8) ^b^

Same small letters indicate nonsignificant results in pairwise comparison. *p* > 0.05 was considered statistically nonsignificant.

## Data Availability

The data used to support this study are available from the corresponding author upon request.
